# *Mycobacterium chelonae* Abscesses Associated with Biomesotherapy, Australia, 2008

**DOI:** 10.3201/eid1909.120898

**Published:** 2013-09

**Authors:** Mihaela Ivan, Craig Dancer, Ann P. Koehler, Michaela Hobby, Chris Lease

**Affiliations:** South Australia Department of Health, Adelaide, South Australia, Australia (M. Ivan, A. P. Koehler, M. Hobby, C. Lease);; SA Pathology, Adelaide (C. Dancer)

**Keywords:** Mycobacterium chelonae, skin abscess, biomesotherapy, alternative therapy, rare pathogens, outbreak, Australia, bacteria, mycobacterium, rapid growing mycobacterium, infection control

## Abstract

An outbreak of skin abscesses occurred in Adelaide, Australia, in association with biomesotherapy, an alternative therapy practice. *Mycobacterium chelonae* was identified in 8 patient and 3 environmental samples. Our findings show *M. chelonae* infection can be associated with alternative therapies when infection-control breaches occur. Tighter regulations of alternative therapy practices are needed.

Biomesotherapy is marketed in Australia as a new therapy that combines homotoxicology, mesotherapy, and acupuncture. Saline solution and homeopathic formulations are injected subcutaneously at specific acupuncture or trigger points, and homeopathic formulations are administered orally during treatment sessions. Biomesotherapy is used for pain management and general well-being.

*Mycobacterium chelonae* is a rapid-growing mycobacterium that occurs naturally in water sources and produces rare infections in humans. The bacterium can cause pneumonia and skin and ocular lesions, mostly following tissue trauma (*1*). *M. chelonae* is commonly present in tap water; however, it is not reliably removed by filtration or by boiling for short periods. Outbreaks and isolated cases of cutaneous infections caused by *M. chelonae* and other rapid-growing mycobacteria have been described in association with alternative therapies, hospital settings, and spas (*2**–**8*).

Mycobacterial infections are notifiable in South Australia, a state in the southern central part of Australia. On average, 2 cases of nonpulmonary *M. chelonae* infection are reported in South Australia each year.

## The Study

In June 2008, the Communicable Disease Control Branch of the SA Department of Health received notification of 5 suspected and 1 laboratory-confirmed case of mycobacterial skin infection. All case-patients reported having received biomesotherapy treatment from the same person, Practitioner A.

An investigation team immediately inspected Practitioner A’s premises and found poor sanitation and infection-control practices in place. Equipment used for injections at the clinic was seized, and Practitioner A was directed to cease all practices related to injection of clients, including the preparation of formulations. A media release and a public health alert were issued to inform the community about the outbreak.

Case finding was then initiated. A case-patient was defined as a person with “skin lesions compatible with mycobacterial infection at injection site and visit to Practitioner A since January 1, 2008.” A list of clients was assembled from Practitioner A’s patient records and appointment book, direct information from patients, and physician referrals. To identify possible case-patients and to collect relevant epidemiologic data, we developed a semistructured questionnaire for use during telephone interviews with Practitioner A’s clients; 43 clients completed the interview. At the end of interviews, clients were strongly advised to consult a general practitioner for clinical assessment and referral to an infectious diseases physician if they had skin lesions or other concerns. 

**Figure 1:**
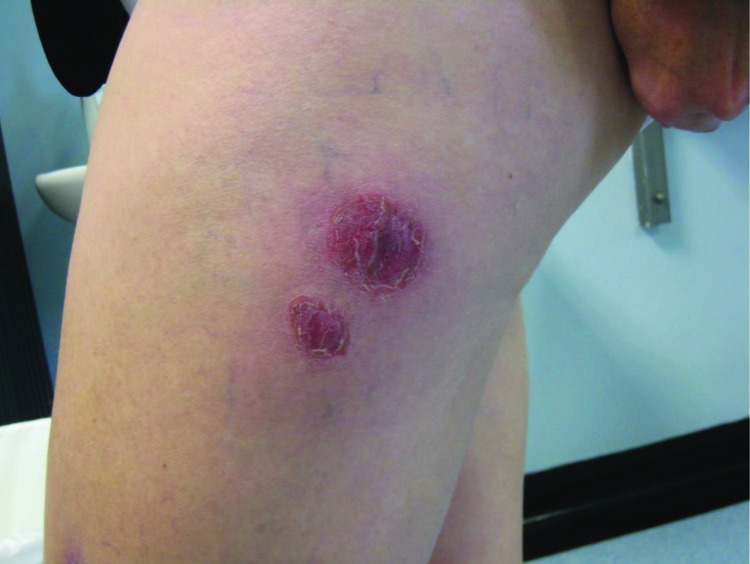
*Mycobacterium chelonae* abscesses associated with biomesotherapy, an alternative therapy practice, Adelaide, South Australia, Australia, 2008. The abscesses are at the biomesotherapy injection site. (Photo courtesy of Erina Gray.)

Through telephone interviews and doctor and laboratory notifications, we identified 27 case-patients, of whom 20 had completed the telephone interview. Abscesses (1–16/case-patient) had developed within days to several weeks after case-patients received biomesotherapy injections from Practitioner A (Figure 1). The mean age of case-patients was 47 years (range 27–77); 17 were female and 20 were male. Thirteen case-patients had visited a physician for their skin lesions, and 18 still had abscesses present at the time of the interview. Twelve case-patients reported that the same needle was used for all injections during a single treatment session. Three case-patients recalled that Practitioner A had washed his/her hands before the procedure; most other case-patients were unsure if this step had been taken.

Twenty clinical samples from 14 case-patients and 36 environmental samples were sent for laboratory testing. The samples were stained (Gram and Ziehl-Neelsen stains) and cultured at 30°C and 35°C on routine agar media in MGIT Mycobacteria Growth Indicator Tube (Becton Dickinson Microbiology Systems, North Ryde, NSW, Australia) broth culture and on Lowenstein-Jensen slopes. The environmental samples were centrifuged and processed as described (*9*). We initially identified organisms by conventional phenotypic methods (*10*). Molecular identification was performed on all isolates by sequencing 3 regions: 16S rDNA, 16S-23S rRNA internal transcribed spacer, and *rpoB* (*11**–**13*). Susceptibility testing was performed by using the disk diffusion test and Etest (bioMérieux, Baulkham Hills, NSW, Australia) (*14*).

Histologic examination of clinical samples revealed inflammation and granulomata, and Ziehl-Neelsen staining of samples from 3 case-patients revealed acid-fast bacilli. Phenotypic testing of isolates showed that arylsulfatase activity was positive at day 3 (weakly positive in 1 isolate); *p*-aminosalicylic acid degradation was positive, but the organism was also tolerant to 5% sodium chloride (results uncertain for 1 isolate); and iron uptake was positive, all of which are suggestive of *Mycobacterium abscessus*. Molecular sequencing identified *M. chelonae* with identical genetic profiles in 8 patient samples and 3 therapeutic solutions, including a Coley toxin homeopathic formulation, from Practitioner A’s clinic. Isolates were susceptible to clarithromycin, amikacin, tobramycin, and tigecycline but resistant to cefoxitin.

## Conclusions

**Figure 2:**
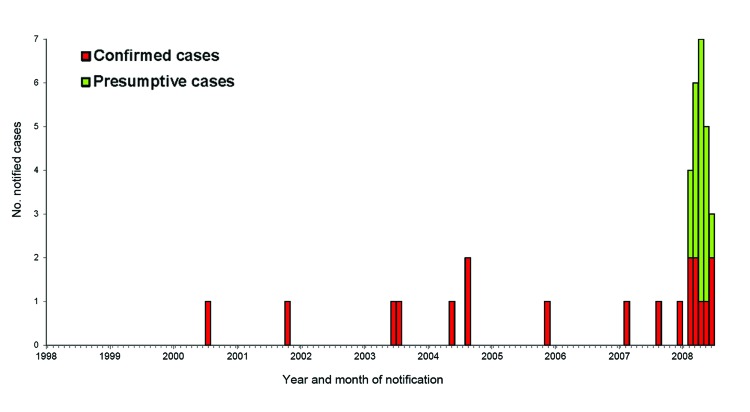
Confirmed and presumptive cases of *Mycobacterium chelonae* infection in South Australia, Australia, by month of onset (January 1998–August 2008). Two presumptive cases from 2008 are not included because onset dates were not known.

These 27 cases of a rare infection occurred in clients of Practitioner A while no other cases were reported elsewhere in South Australia, representing overwhelming epidemiologic evidence that the source of infection was Practitioner A’s clinic (Figure 2). Case-patients had received injections of an *M. chelonae*–contaminated Coley toxin formulation, and the same formulation had been included with colloidal silver in a spray solution that was used to “clean” patients’ skin before injections were administered. These 2 uses of the contaminated formulation constitute plausible routes of infection and the source of the outbreak. Practitioner A’s procedures demonstrated a profound lack of sterile injection techniques and infection-control practices. Therapeutic formulations were kept for long periods in nonsterile bottles that had been washed in tap water; this could have created favorable conditions for *M. chelonae* to multiply in the resulting biofilm. Sterile saline solution was used for the injections; however, it could have been contaminated before use by Practitioner A’s usual procedure of decanting the sterile saline into other containers (e.g., plastic cups) before injection.

Our findings show that *M. chelonae* infection can be associated with alternative therapies if infection-control breaches occur and that tighter regulation of alternative therapy practices is needed. Effective control measures were implemented early in this outbreak; however, the investigation faced many challenges. First, Practitioner A’s client records were incomplete, making it difficult to identify and contact all clients. Furthermore, some persons who use alternative therapies distrust mainstream medicine, so many of the clients were reluctant to be interviewed and/or undergo clinical assessment. Practitioner A also advised clients that an inflammatory reaction (boil) at the inoculation sites was expected as a sign that the biomesotherapy “worked.” This advice prevented or delayed many of the case-patients from seeking medical attention when the skin lesions developed.

Second, most interviewees had poor recollection of the date of symptom onset and the materials and techniques used during treatment sessions. Therefore the interviews did not provide reliable evidence regarding the cause of outbreak. Third, mycobacterial infections are rare and difficult to diagnose. In some case, diagnosis, treatment, and reporting of cases were delayed because patients were referred for assessment by a specialist only after multiple courses of antimicrobial drug treatment failed to resolve symptoms. Case-patients who agreed to clinical assessment and treatment represented a challenge for clinicians because they required prolonged therapy and continuous monitoring for antimicrobial drug resistance. Fourth, the investigation and clinical care of case-patients involved substantial costs to the health care system. 

Last, in the absence of specific legislation governing the practice of biomesotherapy, the regulatory response to this outbreak was undertaken by using general powers under the Public and Environmental Health Act 1987. This was justifiable on the basis of evidence collected, the risk to the public, and the strong likelihood that the harmful practice would continue because of Practitioner A’s lack of insight into his/her role in the outbreak. However the longer term issue of regulating similar practitioners and their practices was also identified as a concern. Outbreaks associated with alternative therapies have sparked calls locally and internationally for the imposition of stringent regulations, including administrative mechanisms requiring registration or licensing of practitioners and mandating minimum standards with regard to premises and procedures.

## References

[1] Mandell GL, Douglas RG, Bennett JE. Principles and practice of infectious diseases. 6th ed. Philadelphia: Elsevier Churchill Livingstone; 2005. p. 2909–16.

[2] Beer K, Waibel J. Disfiguring scarring following mesotherapy-associated *Mycobacterium cosmeticum* infection. J Drugs Dermatol. 2009;8:391–3 . 19363858

[3] Carbonne A, Brossier F, Arnaud I, Bougmiza I, Caumes E, Meningaud JP, Outbreak of nontuberculous mycobacterial subcutaneous infections related to multiple mesotherapy injections. J Clin Microbiol. 2009;47:1961–4. 10.1128/JCM.00196-09 19386853 PMC2691096

[4] del Castillo M, Palmero D, Lopez B, Paul R, Ritacco V, Bonvehi P, Mesotherapy-associated outbreak caused by *Mycobacterium immunogenum.* Emerg Infect Dis. 2009;15:357–9. 10.3201/eid1502.081125 19193300

[5] Garcia-Navarro X, Barnadas MA, Dalmau J, Coll P, Gurgui M, Alomar A. *Mycobacterium abscessus* infection secondary to mesotherapy. Clin Exp Dermatol. 2008;33:658–9. 10.1111/j.1365-2230.2008.02869.x 18616719

[6] Meyers H, Brown-Elliott BA, Moore D, Curry J, Truong C, Zhang Y, An outbreak of *Mycobacterium chelonae* infection following liposuction. Clin Infect Dis. 2002;34:1500–7. 10.1086/340399 12015697

[7] Safranek TJ, Jarvis WR, Carson LA, Cusick LB, Bland LA, Swenson JM, *Mycobacterium chelonae* wound infections after plastic surgery employing contaminated gentian violet skin-marking solution. N Engl J Med. 1987;317:197–201. 10.1056/NEJM198707233170403 3600710

[8] Cooksey RC, de Waard JH, Yakrus MA, Rivera I, Chopite M, Toney SR, *Mycobacterium cosmeticum* sp. nov., a novel rapidly growing species isolated from a cosmetic infection and from a nail salon. Int J Syst Evol Microbiol. 2004;54:2385–91. 10.1099/ijs.0.63238-0 15545488

[9] Lumb R, Stapledon R, Scroop A, Bond P, Cunliffe D, Goodwin A, Investigation of spa pools associated with lung disorders caused by *Mycobacterium avium* complex in immunocompetent adults. Appl Environ Microbiol. 2004;70:4906–10. 10.1128/AEM.70.8.4906-4910.2004 15294830 PMC492441

[10] Brown-Elliott B, Wallace RJ. Clinical and taxonomic status of pathogenic nonpigmented or late-pigmenting rapidly growing mycobacteria. Clin Microbiol Rev. 2002;15:716–46. 10.1128/CMR.15.4.716-746.2002 12364376 PMC126856

[11] Lumb R, Goodwin A, Ratcliff R, Stapledon R, Holland A, Bastian I. Phenotypic and molecular characterization of three clinical isolates of *Mycobacterium interjectum.* J Clin Microbiol. 1997;35:2782–5 . 9350733 10.1128/jcm.35.11.2782-2785.1997 PMC230061

[12] Khan IU, Selvaraju SB, Yadav JS. Method for rapid identification and differentiation of the species of the *Mycobacterium chelonae* complex based on 16S–23S rRNA gene internal transcribed spacer PCR-restriction analysis. J Clin Microbiol. 2005;43:4466–72. 10.1128/JCM.43.9.4466-4472.2005 16145093 PMC1234067

[13] Adékambi T, Colson P, Drancourt M. rpoB-based identification of nonpigmented and late-pigmenting rapidly growing mycobacteria. J Clin Microbiol. 2003;41:5699–708 . 10.1128/JCM.41.12.5699-5708.2003 14662964 PMC308974

[14] Wallace RJ, Dalovisio J, Pankey G. Disk diffusion testing of susceptibility of *Mycobacterium fortuitum* and *Mycobacterium chelonei* to antibacterial agents. Antimicrob Agents Chemother. 1979;16:611–4 . 10.1128/AAC.16.5.611 526002 PMC352914

